# Salt Stress Affects the Redox Status of Arabidopsis Root Meristems

**DOI:** 10.3389/fpls.2016.00081

**Published:** 2016-02-08

**Authors:** Keni Jiang, Jacob Moe-Lange, Lauriane Hennet, Lewis J. Feldman

**Affiliations:** ^1^Department of Plant and Microbial Biology, University of California, BerkeleyBerkeley, CA, USA; ^2^Department of Biology, Stanford UniversityStanford, CA, USA

**Keywords:** redox, auxin, root, meristem, Arabidopsis, salt-stress

## Abstract

We report the redox status (profiles) for specific populations of cells that comprise the Arabidopsis root tip. For recently germinated, 3–5-day-old seedlings we show that the region of the root tip with the most reduced redox status includes the root cap initials, the quiescent center and the most distal portion of the proximal meristem, and coincides with (overlays) the region of the auxin maximum. As one moves basally, further into the proximal meristem, and depending on the growth conditions, the redox status becomes more oxidized, with a 5–10 mV difference in redox potential between the two borders delimiting the proximal meristem. At the point on the root axis at which cells of the proximal meristem cease division and enter the transition zone, the redox potential levels off, and remains more or less unchanged throughout the transition zone. As cells leave the transition zone and enter the zone of elongation the redox potentials become more oxidized. Treating roots with salt (50, 100, and 150 mM NaCl) results in marked changes in root meristem structure and development, and is preceded by changes in the redox profile, which flattens, and initially becomes more oxidized, with pronounced changes in the redox potentials of the root cap, the root cap initials and the quiescent center. Roots exposed to relatively mild levels of salt (<100 mM) are able to re-establish a normal, pre-salt treatment redox profile 3–6 days after exposure to salt. Coincident with the salt-associated changes in redox profiles are changes in the distribution of auxin transporters (AUX1, PIN1/2), which become more diffuse in their localization. We conclude that salt stress affects root meristem maintenance, in part, through changes in redox and auxin transport.

## Introduction

Increases in soil salinity present a notable challenge to agriculture. As a consequence of human activities, many once fertile lands are becoming less productive due to increased salts. Additionally, alterations in rainfall patterns, often associated with climate change, result in less rainfall and hence less leaching from soils of accumulated salts and minerals. As well, the increasing use of more marginal lands for farming often means that growers have to contend with naturally occurring high levels of salts. In total, the FAO estimates that the accumulation of salts impacts on the agricultural production of over 397 million hectares (FAO, 2005). The effects of salts on plant growth and development are numerous, with the severity dependent on the concentration(s) of the salts, the sensitivity of the crop to the salts, and on the capacity of the plants to tolerate or mitigate the effects of the salts (Munns, 2005). Focusing specifically on the influences of salts on roots, typical salt-related effects include decreases in root elongation (Potters et al., 2007; Bernstein, 2013), changes in root architecture (Julkowska et al., 2014), changes in the root gravity response (“halotropism”; Sun et al., 2008), and changes in root anatomy, including decreases in cell size, a reduction in cell division, and alterations in patterns of differentiation, as, for example, the differentiation of the Casparian strip unusually close to the root meristem (Hajibagheri et al., 1989). The inhibition of root elongation by salt is most often linked to a decrease in the amount of cell expansion in the root elongation zone (Zhong and Läuchli, 1993; West et al., 2004; Bustos et al., 2008). But the final length of a root is not only dependent on cell elongation but also on how many cells are available to elongate. In this regard West et al. (2004) show that the response of roots to salt stress also involves an inhibition of the cell cycle, so that cells comprising the meristem stop dividing at a smaller size, resulting in cells elongating closer to the root tip. As a consequence, root meristems in salt-stressed roots are generally reduced in size and therefore produce fewer cells to elongate (Kurth et al., 1986; Zidan et al., 1990; Azaizeh and Steudle, 1991). In summary, as noted by Bernstein (2013) and others (West et al., 2004), the available evidence suggests that salinity inhibits root growth through a combination of the effects of salts on both cell division and cell elongation.

Much of the work investigating the physiological, biochemical and molecular bases for salt-associated changes in roots has focused on the root meristem (Bernstein, 2013). Evidence points to salinity, in part, affecting meristem structure and function as a consequence of salt-induced changes in the distribution of auxin transport proteins (members of the PIN protein family; Chávez-Avilés et al., 2013), resulting in changes in the patterns of auxin distribution at the root meristem. Recently, Iglesias et al. (2014) advanced the notion that suppression in auxin signaling may be a way in which plants modify (increase) their tolerance to salinity, and these workers showed that this mechanism involved a repression by NaCl of the Aux/IAA repressors. A more general physiological response of roots to salinity is the onset of oxidative stress, characterized by an increase in, and accumulation of various reactive oxygen species (ROS), such as OH- and H_2_O_2_. Toxicity is generally associated with the induction by ROS of a cascade of reactions resulting in the initiation of a variety of physiologically detrimental processes (Bernstein, 2010; Hernandez et al., 2010). With regard to ROS toxicity, much effort has been directed to understanding the antioxidant mechanisms that plants use to mitigate the effects of salinity-induced ROS, with a focus on the salt-stress-induced changes in the activities of antioxidant (ROS-detoxifying) enzymes (Dat et al., 2000; Mittler, 2002; Apel and Hirt, 2004). Not surprisingly, salt-stress-induced changes in the root's antioxidant response are reported to be spatially and temporally variable, and likely also linked to the developmental stage of the root tissues and cell types (Hernandez et al., 2010).

Although ROS production is generally considered to be detrimental, there is convincing evidence that ROS may also have a dual role, and function positively to promote many developmental processes, such as signaling and cell expansion (Rodríguez et al., 2002; Foreman et al., 2003; Rubio et al., 2009; Shoresh et al., 2011). Therefore, maintaining a balance, or homeostasis in ROS is considered central to normal development, as suggested earlier by Kerk and Feldman (1995) and Jiang et al. (2003) for the organization of the root meristem. Thus, based on an appreciation of this dual role for ROS, and on the hypothesis that salt stress causes changes in root ROS homeostasis, an important question to ask is, how is ROS status affected in roots treated with varying levels/periods of exposure to salinity? Surprisingly, little information addresses this central question. Not only are data lacking on the redox status of salt-stressed roots, but as well, little is known of the redox status for non-stressed roots. Using redox sensitive dyes investigators have demonstrated *relative* levels of particular species of reactive oxygen, but these results are mostly qualitative, often specific for one type of reactive oxygen species, and thus not a measure of the overall redox state of the tissue (Shin et al., 2005). Here, we report on the overall redox status in Arabidopsis roots in non-stressed and also in saline environments. We focus on the root meristem (terminal mm of the root) because it is here where many salt-sensitive processes (e.g., cell division and elongation) predominately occur. We report, for the first time, the redox potentials for various regions of the root meristem in relation to imposed salt stress. We show that different regions of the root respond uniquely to salt stress, not only spatially, but temporally as well. Moreover, the differing responses to varying levels of salt point to mechanisms, and to limitations used by roots to adjust (or not), to saline soils. For this effort we employ Arabidopsis seedlings which express a redox-sensitive reporter (roGFP) (Hanson et al., 2004) that we have previously described (Jiang et al., 2006), and which has also been used recently for a determination of redox status in plants exposed to drought stress (Brossa et al., 2013).

## Materials and methods

### Plant material

For measuring and monitoring redox potentials seed of *Arabidopsis thaliana* transgenic line c-roGFP (Jiang et al., 2006) were used. For auxin-related experiments the following Arabidopsis transgenic marker lines were used: *DR5*:*GUS* (Ulmasov et al., 1997), PIN1:GFP (Friml et al., 2002); PIN2:GFP (Xu and Scheres, 2005), AUX1:YFP (Swarup et al., 2004). The expression patterns of each of these promoter lines were visualized using a Zeiss LSM710 confocal microscope. For GUS staining, seedlings were placed in the GUS solution (Ulmasov et al., 1997) for 1 h at 37°C, after which time the roots were fixed (ethanol:acetic acid, 3:1) and then mounted in chloral hydrate.

### Salt-stress treatment

**Figure 1:**
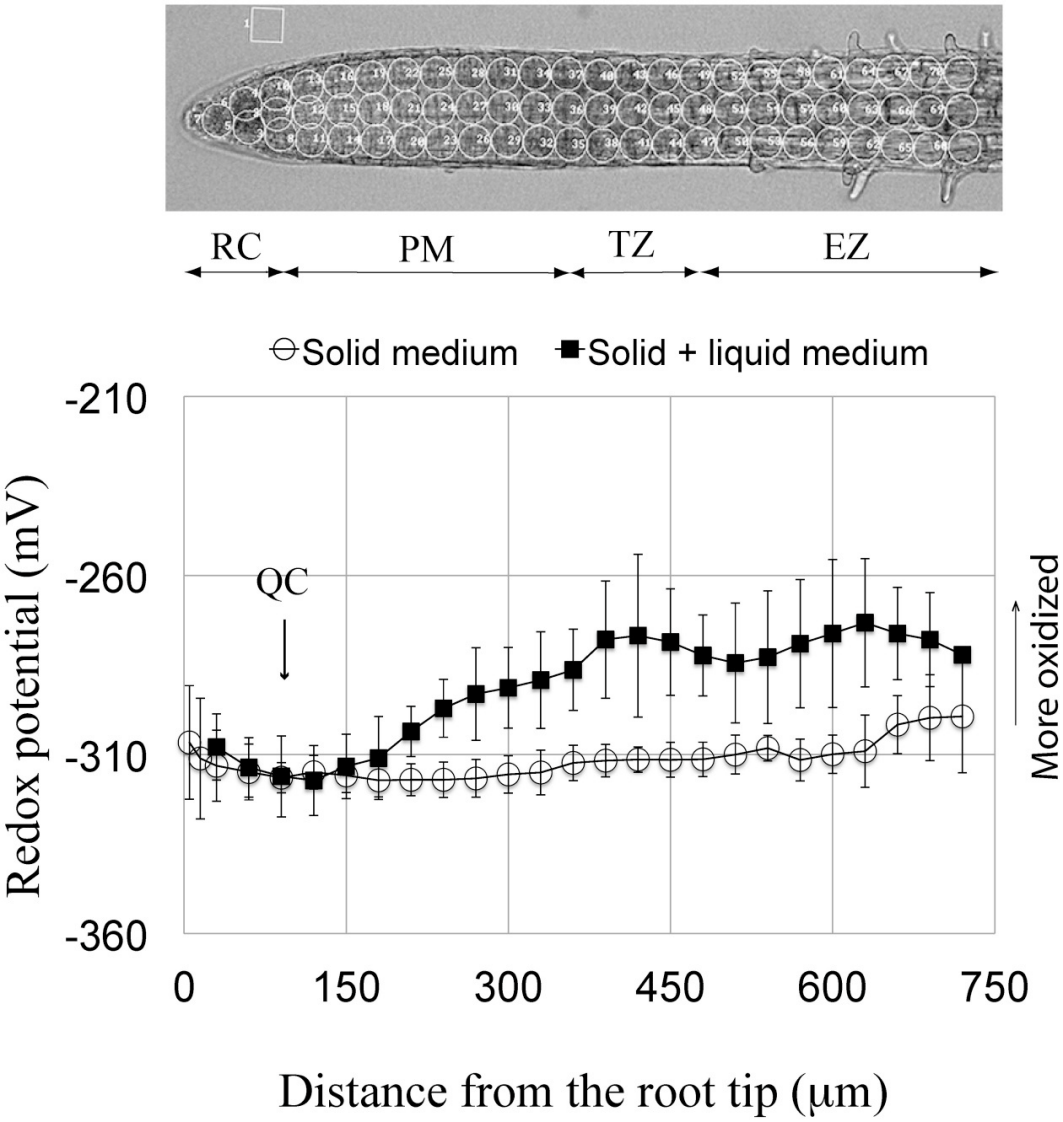
**Redox potential profile of the primary root tip of Arabidopsis**. Circled regions in the root above the profiles indicate areas of the redox measurements. Two profile curves are shown; one from roots grown continuously on solid 0.5x MS agar medium (open circles) and the other (solid squares) shows the redox profile from roots grown for 5 days on 0.5x MS agar and then immersed in 0.5x MS liquid medium for 3–24 h. Each curve is a summation obtained by averaging the measurements from the individual, corresponding curves shown in Supplementary Figure 1 (i.e., for immersion, averaging control curves from 3, 6, 12, and 24 h, and for growth on solid medium only, averaging the control curves from 3, 6, and 9 days). Standard deviations are shown for the 48 roots used to obtain the redox profile for roots grown continuously on agar, and for the eight roots used to obtain the redox profile for roots grown on agar with subsequent immersion. The more negative the potential, the more reduced is the redox status. Note that the two curves overlap in the distal-most 180 μm of the root. RC, root cap; QC, quiescent center; PM, proximal meristem; TZ, transition zone; EZ, elongation zone.

Seeds expressing the c-roGFP (Jiang et al., 2006) were surface sterilized and plated on 0.5x MS agar (+1% sucrose) supplemented with varying concentrations of NaCl (0, 50, 100, and 150 mM) and then incubated for 3 days at 4°C, following which the plates were transferred to a growth chamber (16 h photoperiod, 23.5°C). After varying periods of time (3–9 days) on the NaCl-supplemented agar media, the vertically-oriented Petri dishes on which the seedlings were growing were re-oriented to the horizontal and placed directly on the stage of the microscope. A coverslip was affixed and measurements made of the redox status of the root meristem. For each time point (i.e., 3, 6, and 9 days) *n* = 16. The controls for all three time points (*n* = 48) are averaged to give an overall control plot as seen in Figure 1. (Supplementary Figure 1A shows the three individual plots used to obtain the average shown in Figure 1). In order to assess the effects of short periods of exposure to salt (3, 6, 12, and 24 h), the experimental set-up had to be modified so that roots could reach a developmental stage suitable for observation (at least 3–5 days after germination). For these short time-exposures, salt-stress experiments seeds were sterilized and plated on salt-free 0.5x MS medium supplemented with 1% sucrose, incubated in cold for 3 days, and allowed to germinate with the Petri plate oriented vertically for an additional 5 days (16 h photoperiod) at 23.5°C. Subsequently the vertically-oriented plates were immersed in 150 mM NaCl in 0.5x MS (no sugar) so that only the roots were submerged in the salt-supplemented liquid medium. After a 3 h exposure to salt the redox status of the root, which remained attached to the agar medium, was measured. After each measurement the Petri dish was returned to the vertical position and re-immersed in the same liquid salt solution, as before. Redox measurements were again made on the *same* roots after 6, 12, and 24 h additional exposure to salt. After the final measurement (at 24 h) the roots were treated with H_2_O_2_ followed by DTT, in order to normalize for calculating redox potential (as described in Jiang et al., 2006). A control curve, for short-term exposure to salt (Figure 1) was obtained by averaging the four individual control plots (immersion in 0.5x MS only) for 3, 6, 12, and 24 h (*n* = 8 for each time point; Supplementary Figure 1B).

### Ratiometric measurements of redox status

Ratiometric measurements were made as described previously (Jubany-Mari et al., 2010). Briefly, a Nikon Diaphot FN600 microscope fitted with Plan Fluor 10x/0.30NA dry objective and a Chroma Technology Phluorin filter set (exciters D410/30 and D470/20, dichroic mirror 500DCXR, emitter HQ535/50) microscope was used. Images were captured of the entire terminal mm of the root using a Hamamatsu Orca-100 cooled CCD camera (Hamamatsu Corp.). Emission data (fluorescence) were collected over time and processed using MetaFluor 6.1 image analysis software (Molecular Devices), which controlled both filters and the data collection, as detailed by Jiang et al. (2006).

Measurements of redox status were performed by focusing on the median plane of intact roots, which raises the question of whether the redox values measured in this manner provides accurate readings of median plane redox status. By acquiring a “z” stack of optical sections using a Zeiss LSM710 confocal microscope, equipped with a 20x objective, we were able to confirm that redox potentials measured using the aforementioned Nikon microscope set-up accurately reflects redox status as measured using a confocal microscope focused on the median plane (data not shown).

### Root anatomy; measurements of internal cellular characteristics

After various times on the salt-supplemented media root lengths were measured (distance between the hypocotyl junction and the root tip). Subsequently, roots were fixed for microscopy (3:1, ethanol:acetic acid) at 4°C, for 1 week, and for observations mounted in chloral hydrate. Cell counts and cell length measurements were obtained from cortical files in reference to landmarks (quiescent center [QC], elongation zone [EZ]; Perilli and Sabatini, 2010). The length of the proximal meristem (PM), defined by Verbelen et al. (2006) as the “root meristem,” included cells between the QC and the first isodiametric cell, which marked the distal end of the transition zone (TZ region). The TZ includes all isodiametric cortical cells between the PM and the EZ (the EZ is defined as originating at the point [location] in the root at which cells lose their isodiametric shape and begin to elongate). After mounting in chloral hydrate roots were photographed and cell shapes and lengths analyzed using ImageJ software. At least six roots were used for each measurement and data were analyzed using the Student's *t*-test and *p*-values determined. For our work, results were considered to be statistically significant with a *p* < 0.05.

## Results

### Redox profiles of control root tips

Here we characterize, for the first time, the overall redox status of specific zones comprising the terminal mm of the Arabidopsis primary root. We report that the redox status/profile can vary, depending on whether seedlings are grown on agar only, or on agar plus short-term immersion in liquid culture medium. But irrespective how seedlings were grown, the root cap (RC) shows a relatively oxidized redox status (−308 to −312 mV), and that as one moves in a proximal direction, the overall redox level gradually becomes more negative (more reduced), so that the redox state of the QC is at, or nearly at, the most reduced redox level of any cells at the root tip (−317 mV; Figure 1, Supplementary Figure 1). Just proximal to the QC begins the zone of maximum cell divisions (the proximal meristem [PM]). Cells of the PM directly adjacent to the QC show a similar or slightly more reduced redox status compared to their neighboring QC cells. Moving more proximally in the PM, and focusing on seedlings grown long-term on agar, we observe a rise in the overall redox state, indicating a more oxidized status, which is reflected by a change in redox potential between the distal and proximal boundaries of the PM. Moving further proximally into the TZ, the redox status plateaus and levels off at an average redox potential of about −312 mV. Continuing to move basally from the TZ into the EZ the redox potential initially is little changed and fluctuates somewhat around −310 mV. With further movement proximally the redox potential of the EZ becomes markedly more positive (more oxidized) reaching a value of about −300 mV at a distance of about 750 μm from the root tip. This very reproducible redox profile, for different-aged roots grown on plain agar, contrasts markedly with the profile of roots grown 5 days on agar, and subsequently immersed in a bathing solution (Figure 1). Short-term immersion of roots results in a remarkably different, reproducible redox profile, compared to roots grown solely on agar. For roots immersed 3–24 h the redox status of the PM becomes markedly more oxidized, beginning ~100 μm proximal to the QC/PM border, reaching a value of about −280 mV at the PM/TZ interface. As the only difference in environments leading to these contrasting profiles is, whether or not seedling roots are or are not immersed, we attribute the more oxidized status of immersed roots as due to the onset of immersion-induced oxidative stress in the portion of the terminal mm beyond the most-distal 180 μm, a point to which we will return in the Discussion.

### Redox profiles in tips of salt-treated roots

**Figure 2:**
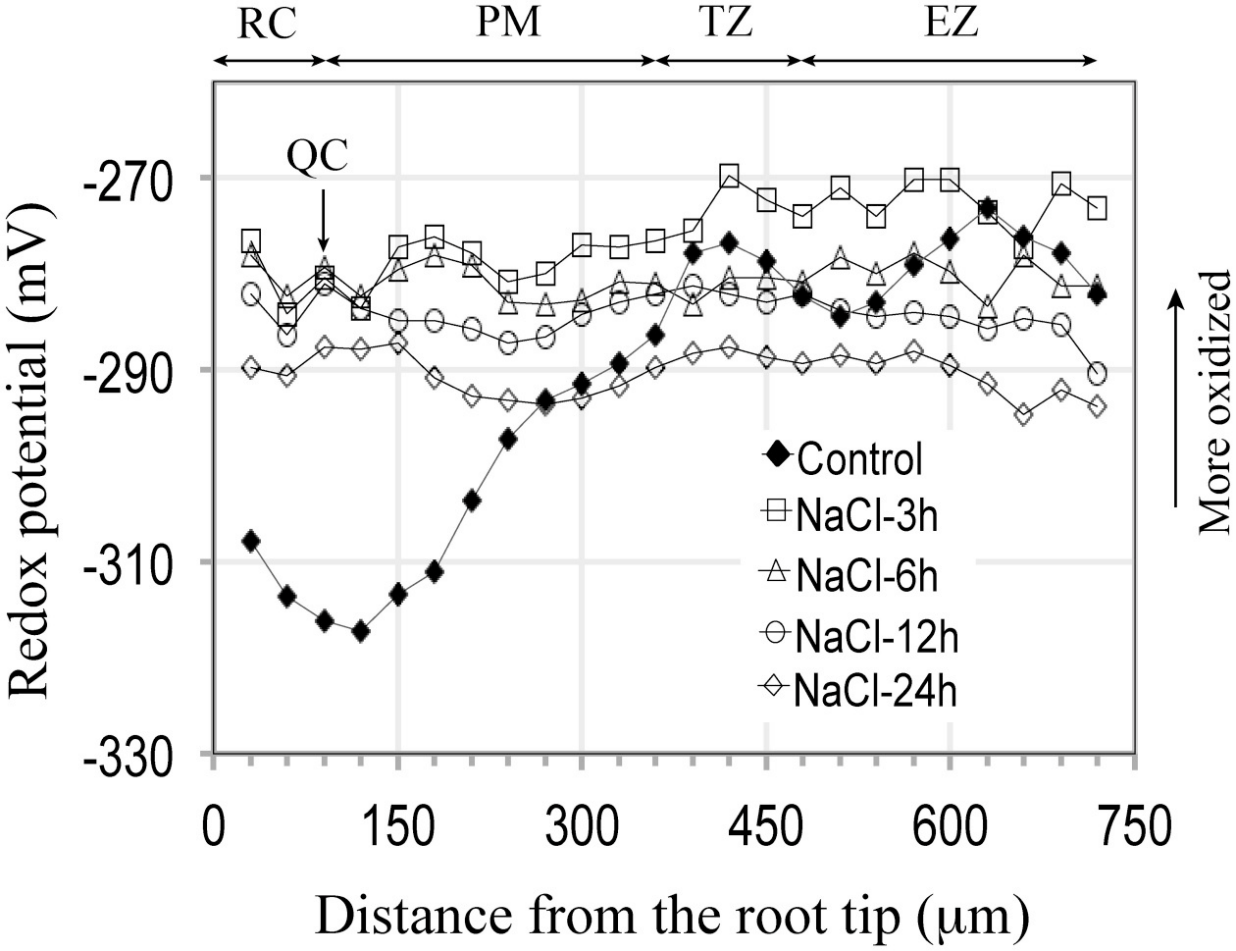
**Redox potential profiles of Arabidopsis primary roots grown 5 days on soild agar and then immersed in 100 mM NaCl for varying periods of time (3–24 h)**. For all periods of exposure the redox status of the entire root becomes more oxidized, with the greatest change occurring in the terminal 300 μm (Compared to control curve from Figure 1). Each salt treatment redox profile curve represents an average from 7 to 8 roots, for which the standard deviations are shown in Supplementary Figure 2.

**Figure 3:**
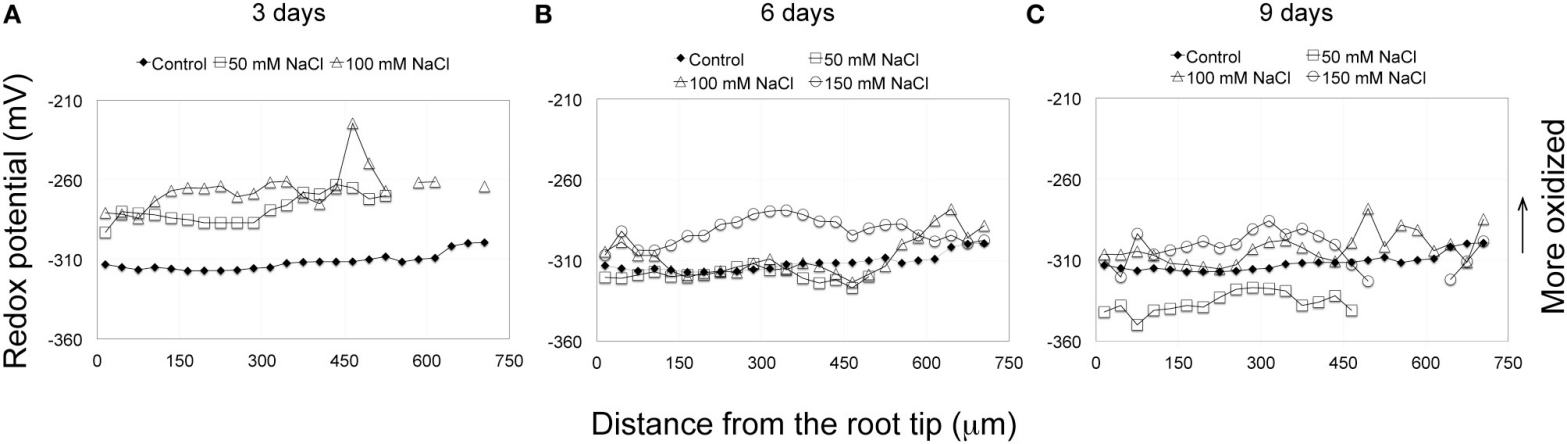
**(A–C)** Redox profiles of Arabidopsis primary roots, grown on solid medium, and treated for extended periods (3–9 days) with various concentrations of NaCl (50–150 mM). After 6 days treatment with 50 mM NaCl the redox profile more or less matches the redox values and profiles of the control, whereas by day 9 the overall redox status of 50 mM salt-treated roots has become more reduced than the control. Except for the terminal 180 μm, the redox profile of 100 mM salt-treated roots matches the control, but by day 9 becomes, overall, more oxidized. The redox profiles of roots treated continuously with 150 mM NaCl, in general, remain at values more positive (more oxidized) than the control. The control curve (solid squares) is obtained as described in Figure 1. Each salt treatment redox profile curve represents an average from eight roots, for which the standard deviations are shown in Supplementary Figure 3.

Roots were exposed to varying concentrations of salt (50, 100, and 150 mM NaCl) for different lengths of time and the redox status of the root tip characterized. Following short-term treatments (immersion) for 3, 6, and 12 h with 150 mM salt, the overall redox status of the terminal 180 μm (RC, QC, and distal PM) has become more oxidized (Figure 2, Supplementary Figure 2). After a 24 h salt treatment the redox status has begun to shift to a more negative (reduced) redox state. With increasing periods of exposure (6 and 9 days) to salt, the redox potentials move to more negative (reduced) redox potentials, shifting from values between −270 and −290 mV, to values between −280 to −310 mV (Figures 3A–C, Supplementary Figure 3). With the exception of the QC, exposing roots for 3 days to 100 mM salt, results in a more oxidized redox state compared to controls. However, extending the salt treatment to 6 days, again with the exception of the QC, results in an overall redox pattern matching that of control roots. But after a 9-day, 100 mM salt treatment the overall redox status of the root becomes slightly more oxidized compared to the control. Interestingly, the region of the QC shows little change from a 6-day salt treatment and remains as the most oxidized zone of the 9 day, 100 mM salt-treated roots. Treating roots with 50 mM salt, the lowest NaCl concentration used, showed a 3 and 6 day redox response pattern quite similar to exposure of roots to 100 mM salt, with an initial rise (indicating a more oxidized status), and by day 6 a return to a more reduced redox status, closely matching the control. However, after 9 days of exposure to 50 mM salt, unlike a 9-day exposure to 100 and 150 mM salt, the redox status becomes even more reduced and overall, the redox profile becomes even more negative (more reduced) than the control (Figure 3C, Supplementary Figures 3C,F,H).

### Effects of salt treatments on overall root length, cell number, and cell length

**Figure 4:**
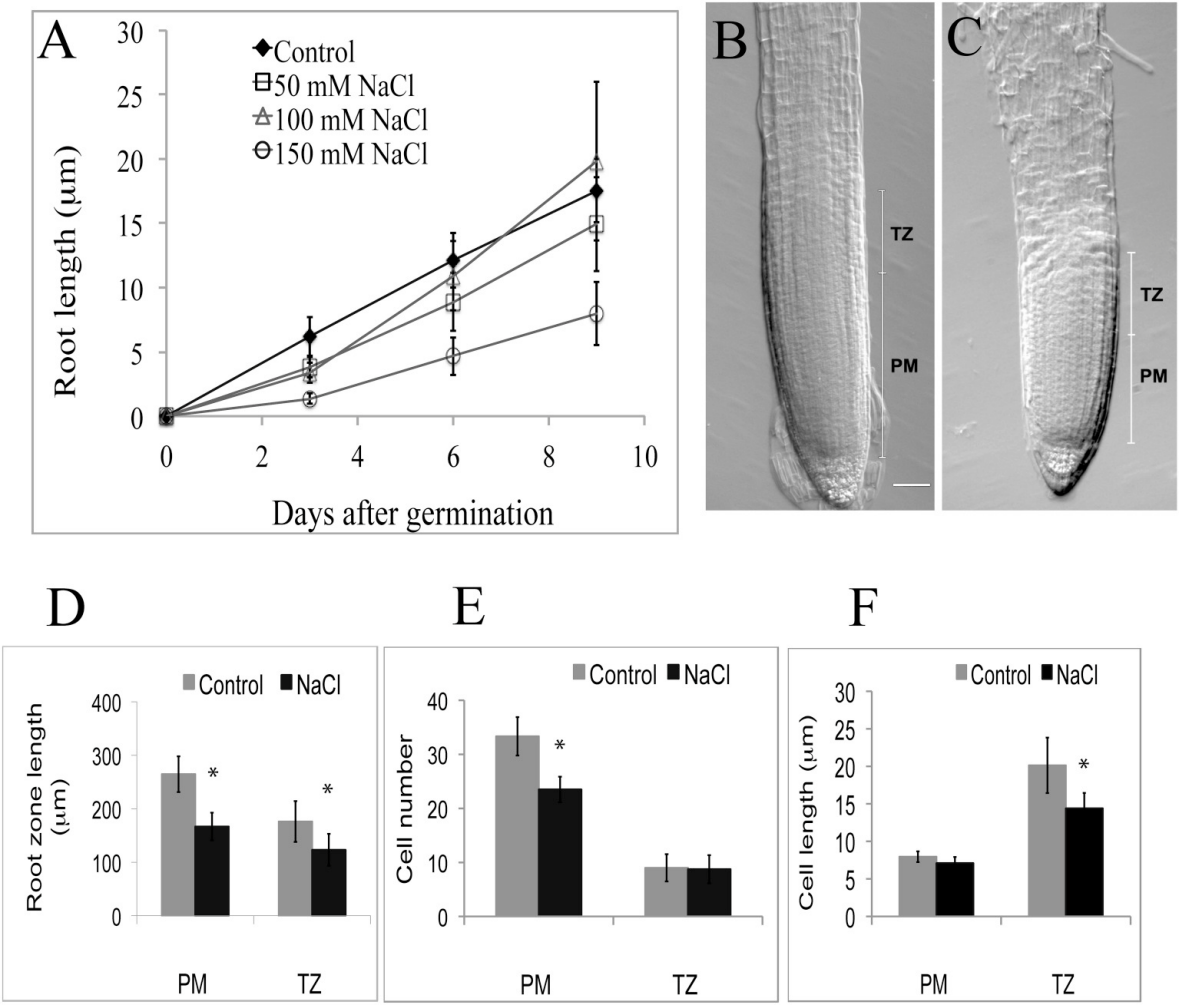
**(A–F)** Growth parameters of the proximal meristem and transition zone in Arabidopsis primary roots, ± salt treatments. **(A)** Root length after varying periods of treatment with NaCl (50–150 mM). **(B)** Intact 3-day-old control root tip. **(C)** Intact tip from root treated 3 days with 100 mM NaCl. Note the shortening of the PM in the salt-treated root (scale bar = 20 μm). **(D–F)** Comparison of cellular characteristics of the PM and TZ in roots treated with 100 mM salt for 3 days: D, overall lengths; E, number of cells; F, cell length. PM, proximal meristem, TZ, transition zone, *n* = 6; ^*^indicates a statistical significance of *p* < 0.05.

Salt treatments affect root length (Figure 4A). After a 3-day treatment (50, 100, and 150 mM NaCl) roots are statistically (*p* < 0.05; *n* = 19) shorter than the control. Focusing specifically on the terminal half mm region in 100 mM salt treatments, we report that all reduction in length is due to a statistically significant (*p* < 0.05; *n* = 6) reduction in the length of the PM (Figures 4B–D), which is linked to a reduction in cell number in the PM of salt-treated roots, compared to controls (Figure 4E), and to a slight, but statistically significant reduction in cell length in the TZ (Figure 4F). On average, cell numbers in the PM of salt-treated roots declined by 29%, with no effect of salt (100 mM) on cell length (Figures 4E,F), whereas for the TZ, only cell length, but not cell number was affected by the salt treatment (Figures 4E,F). Thus, salt-treatment affected the functioning of both the PM and the TZ, but in different ways.

### Effects of salt treatments on auxin distribution and on the localization of auxin transporters

**Figure 5:**
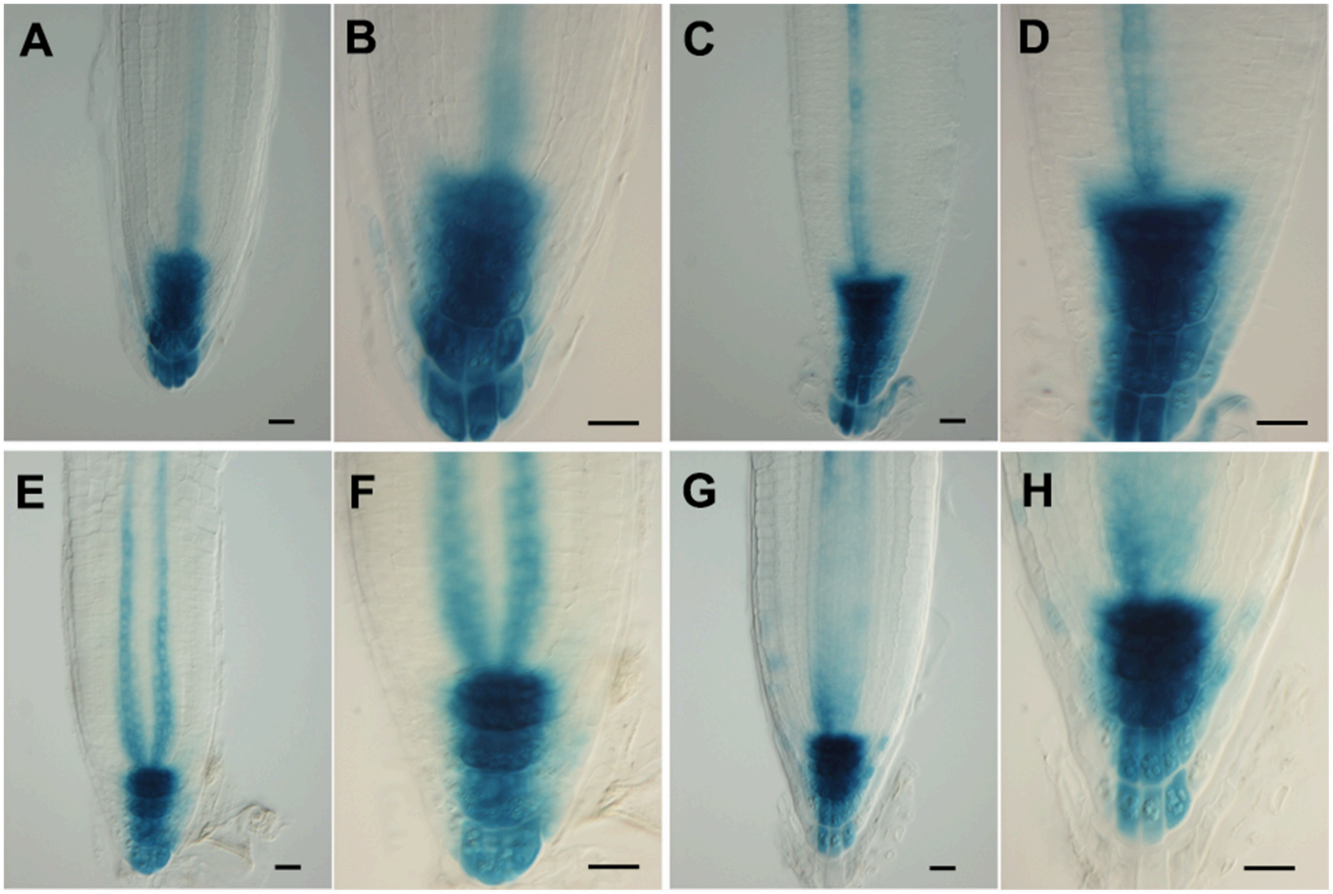
**(A–H)** Patterns of expression of an auxin-responsive promoter (DR5) in Arabidopsis root tips, ± salt treatment. Using the DR5GUS reporter in roots treated with 150 mM salt for 3 h **(A–D)** or 24 h **(E–H)** in order to reveal auxin distribution. **(A,B,E,F)** control; **(C,D,G,H)** plus salt. Scale bar = 20 μm. *n* = 10 roots for control and for each treatment.

**Figure 6:**
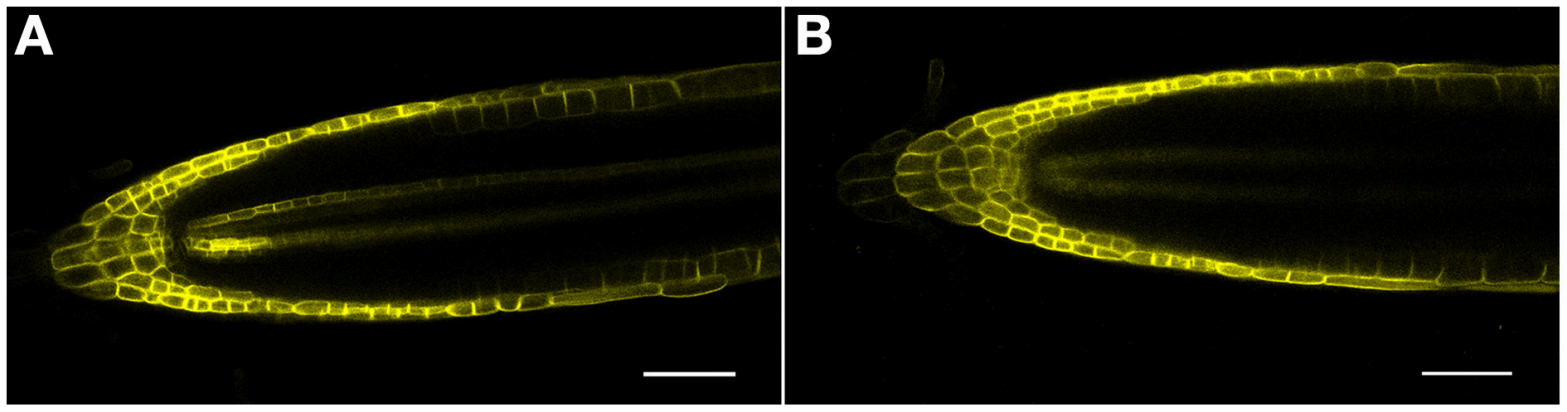
**(A,B)** AUX1 (AUX1-YFP) confocal microscopic localization in roots, ± salt. **(A)** Control root; note reporter expression in both the epidermis and vascular tissue. **(B)** Root treated with 150 mM salt for 6 h. Note the loss of AUX1 expression in the central vascular tissue as well as a diminishment in expression in both the QC and RC. Scale bar = 50 μm, *n* = 4 roots for control and eight roots for each treatment.

**Figure 7:**
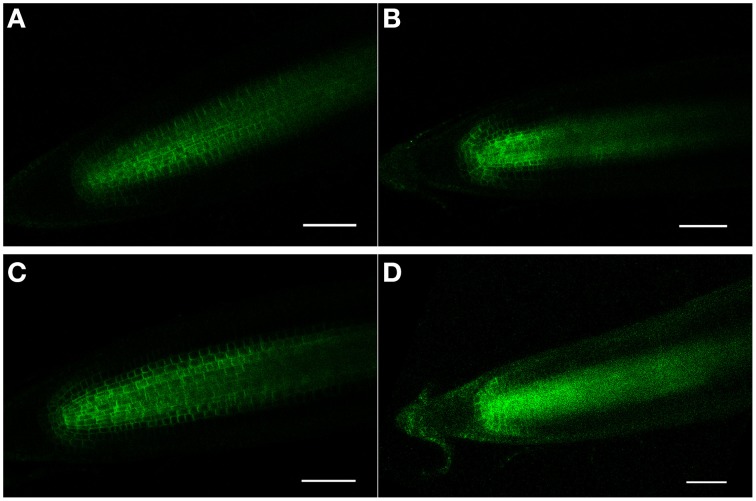
**(A–D)** PIN1 (PIN1-GFP) confocal localization in Arabidopsis roots, ± salt. **(A,C)** Control roots (no salt treatment) after 12 and 24 h, respectively. **(B,D)** Roots treated for 12 and 24 h, respectively, with 150 mM salt. Note that after 24 h of salt treatment that PIN1-GFP expression has become diffuse and is no longer specifically associated with the membranes. Scale bar = 50 μm, *n* = 6 roots for the control and seven roots for each treatment.

**Figure 8:**
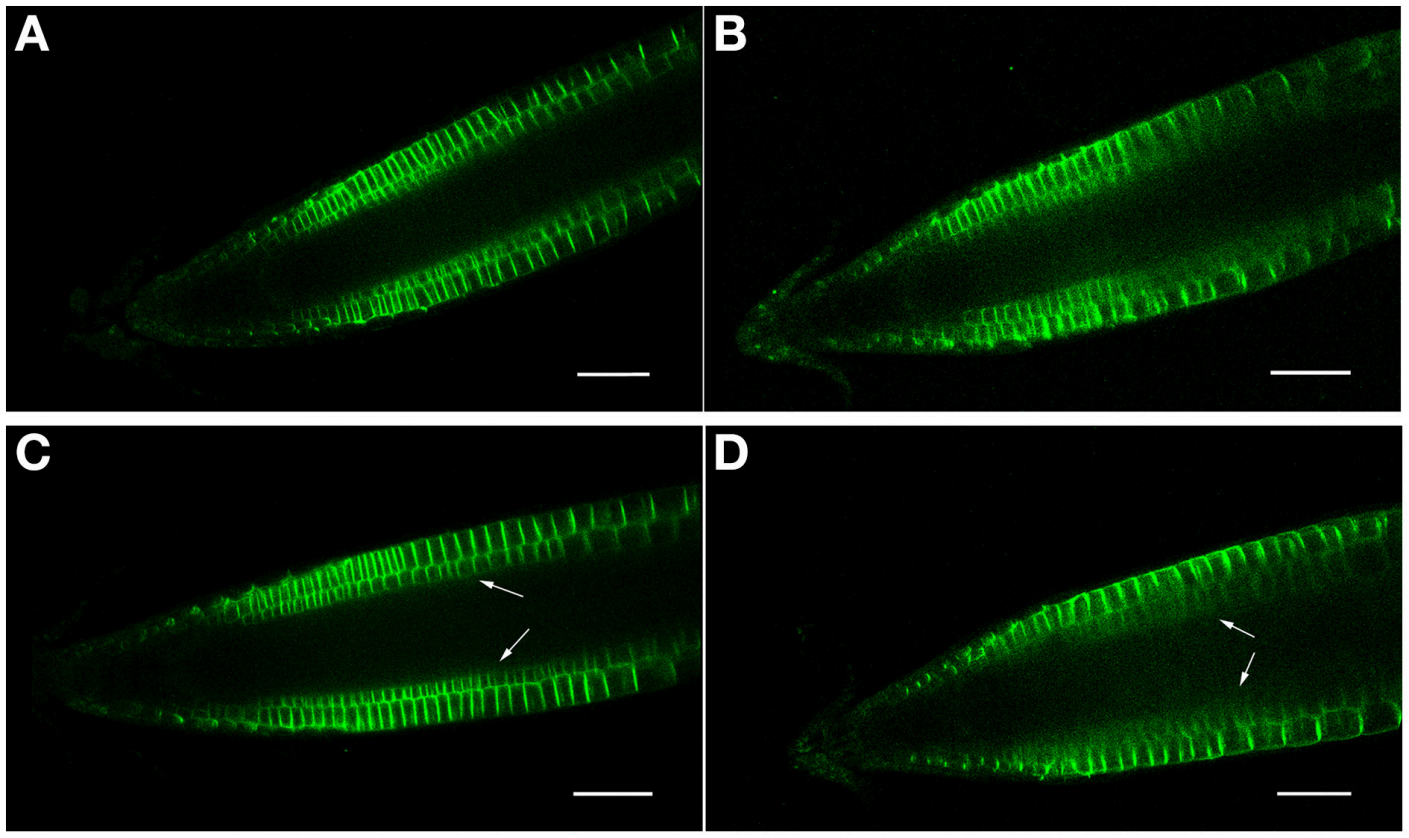
**(A–D)** PIN2 (PIN2-GFP) confocal localization in Arabidopsis roots, ± salt. **(A,C)** Control roots (no salt treatment) after 12 and 24 h, respectively. **(B,D)** Roots treated for 12 and 24 h, respectively, with 150 mM salt. Note that a 24 h salt treatment results in a diminishment of PIN2-GFP expression in the cortical layer directly beneath the epidermis (arrows). Scale bar = 50 μm, *n* = 6 roots for the control and seven for each treatment.

As proposed earlier (Chávez-Avilés et al., 2013), we also observe a modest increase in the expression of an auxin-regulated promoter (*DR5GUS*) following salt treatments, suggesting an increase in the accumulation of auxin at the root tip, with greater staining (higher levels) of auxin found in the regions basal (proximal) to the QC in the root tip in 3 h, 150 mM salt-treated roots, as compared to controls (Figures 5A–H). Salt treatment of roots perturbs the localization of auxin transporters. Following a 6 h salt treatment, AUX1, an auxin influx carrier (Kleine-Vehn et al., 2006), becomes more restricted in its distribution and is no longer expressed in the cells of the vascular tissues, and also shows a diminished level of expression in the RC (Figures 6A,B). Likewise, for both PIN1 and PIN2, auxin efflux carriers (Zazímalová et al., 2007), salt treatment perturbs the distribution of these carriers. For PIN1, a 24 h salt treatment almost completely abolishes any association of the protein with membranes (Figures 7A–D), whereas for PIN2, salt treatment results in a disappearance of the protein from the cortical layer directly to the inside of the epidermis, and as well, in a diminishment (12–24 h after beginning the salt treatment) in the localization of the PIN2 to membranes in the epidermis (Figures 8A–D). But unlike for AUX1 whose pattern is changed by 6 h after beginning the salt treatment, for PIN1/2 a change in pattern is not observed until 10–12 h after beginning salt treatment, considerably after the salt-induced change in redox (at about 3 h).

## Discussion

### The redox profile of the arabidopsis root tip

Arabidopsis root tips display a complex pattern of redox potentials, with the region of the QC and the adjacent cells of the PM showing the most reduced potentials (Figure 1). In comparison, the adjacent RC bordering directly on the distal face of the QC shows a more oxidized status. The complexity in the redox state and pattern at the root tip are influenced by the immediate environment surrounding the roots, as can seen by comparing the redox profiles from control roots grown continuously on solid medium, with the profiles from roots grown initially on solid medium, but then subsequently immersed in the same, but now liquid medium for 3–24 h (Figure 1). For both treatments the redox profiles are identical for the most distal, terminal 180 μm of the root, which includes the RC, the QC and the most distal region of the PM. However, proximal to the most distal 180 μm region of the root the redox profiles diverge, with more oxidized values observed for roots immersed in liquid, as compared to the profiles from roots grown continuously on agar. While the shapes of the two curves are similar (e.g., both show the development of more negative redox potentials as one moves through the PM to the TZ), the magnitude of the response differs between the two treatments. We conclude that immersion, even for short periods (3 h), may induce a level of stress that results in relatively oxidized values for regions of *control* roots proximal to the most distal 180 μm, and that this immersion-related stress masks or overlays possible salt-induced changes in redox potentials. For this reason, possible salt-associated changes in the redox profiles in the regions beyond distal-most 180 μm region of the root tip cannot be determined using immersed roots. But since, immersion does not result in changes of the redox profile within the first 180 μm of the tip, this allows us to explore the effects of short-term exposure to salt on the distal-most region of the root tip (the RC, QC, and distal PM). This also suggests that with regard to redox status that the tip-most region of Arabidopsis roots is either unaffected by immersion stress, or has unique mechanisms to buffer oxidative stress, and therefore to retain a pre-immersion redox profile.

In non-immersed, control roots the PM (the region of highest mitoses) initially exhibits a stable, reduced redox potential (−317 mV) which rises to −312 mV (a more oxidized potential) at the proximal border of the PM, at which point cell divisions have ceased (at the PM/TZ boundary). Redox status has been linked to cell division activity. As early as 1931 Rapkine demonstrated in sea urchin eggs that the oscillating patterns in thiol accumulation could be linked to the cell cycle (Rapkine, 1931). Since this observation, considerable evidence has accumulated pointing to reactive oxygen species (ROS) functioning as second messengers regulating cell proliferation. Thus, it is now generally accepted that oscillations in redox metabolism represent a fundamental mechanism linking oxidative metabolism to the regulation of the cell cycle, and to subsequent differentiation (Sauer et al., 2001; Menon and Goswami, 2007; Fehér et al., 2008; Sarsour et al., 2009). Tsukagoshi et al. (2010) have recently explored the transition from proliferation to differentiation in roots and conclude that there is a “clear correlation between growth rate, location of the TZ, and the relative distribution of different ROS species” in the meristem and in the zone of elongation. At this point it is probably fair to conclude that for Arabidopsis roots, that as the redox status of the main zone of cell division (the PM) becomes more oxidized, that this is associated with a decrease in mitoses and with the onset of differentiation. However, it is also clear that redox status by itself does not determine whether a cell will divide, as demonstrated by the infrequent divisions of cells of the QC, which exhibit redox potentials more reduced than found in the dividing cells of the PM. A consideration of other factors affecting cellular proliferation, besides redox status, will be discussed subsequently.

### Effects of salt treatments on the root redox profile

Being cognizant of the possible effects of immersion on redox profiles, we can, nevertheless conclude that for the most distal 180 μm of the root tip, that salt treatments (3 h, 150 mM) result in marked changes in redox status, shifting the redox potentials of the RC, QC and distal PM by 32, 38, and 30 mV, respectively, to more oxidized values (Figure 2). Increasing the exposure period to salt to 24 h does not result in more oxidized redox potentials within the terminal 180 μm region, compared to a 3 h salt treatment. Thus, we conclude that an initial, short-term (3 h) exposure to salt is sufficient to trigger the maximal change in redox; even prolonging the salt treatments to 3 days does not move the redox profile (values) in a more oxidized direction. With increasing periods of exposure to salt the redox profiles begin to shift from maximum oxidized values (−280 to −270 mV) to more reduced values (−300 to −290 mV) after 6 days of salt treatment, and to even more reduced values after 9 days of salt exposure (−310 to −300 mV), thereby suggesting that in such roots, processes have been initiated to buffer and ameliorate salt-induced changes in redox. Of particular note are the long-term redox responses of roots treated with 50 mM salt, for which we find that after 9 days exposure to salt that the redox profile has, overall, shifted to a level that is about 20 mV *more* reduced than control roots. Fifty mM salt-treated roots, therefore, appear to have overcompensated in the readjustment of their redox profiles in response to salt stress. Our results further suggest that Arabidopsis roots are unable to restore their redox values to control profiles when exposed to salt concentrations between, or exceeding 100 to 150 mM NaCl.

Considerable research now points to plants responding to increased ROS via a variety of mechanisms, most often involving antioxidant enzymes (Bernstein, 2013). Although, beyond the scope of this research, our work suggests that there must be a complex balancing of the redox status along the length of the root, reflecting the roles of ROS in both promoting (e.g., for cell elongation), as well as inhibiting many cellular activities. Perturbing this balance, either chemically of genetically, can affect cell size, as recently demonstrated by Lu et al. (2014) with mutants of Arabidopsis in which the expression of peroxidase genes is upset. For our work treating roots with salt, initially all levels of salt cause a decrease in root length. In this regard it is worth noting that as with unusually oxidized redox levels, unusually reduced levels may also negatively impact on root physiology, as suggested by a reduction in length of 50 mM salt-treated, compared to controls (Figure 4A). After 9 days of salt treatment roots exposed to 100 mM salt exceed the length of control roots, whereas roots treated with either 50 or 150 mM salt are shorter than control roots, suggesting that there is an optimal redox value, neither too reduced (as in 50 mM treatments), nor too oxidized (as in 150 mM treatments) at which root elongation is optimal (Figure 4A). West et al. (2004) explored the cellular basis of these differences in lengths in salt-treated Arabidopsis roots and concluded that salt did not affect the length of the cell cycle, but rather that salt reduced overall root length by affecting meristem size and final cell length. Our results support the conclusion that high levels of salt (>100 mM) cause a decrease in the size (number of cells) in the PM (Figures 4A–F), the primary region of production of new cells for the root. Here we suggest that the size of the PM, and its functioning, are related to an overlaying of the redox gradient, so that maximum cell divisions occur within a region of the PM that is neither too oxidized, nor too reduced in redox potential. It is, we suggest, no coincidence that the PM/TZ boundary is marked by a reduction/cessation in cell division that is associated with a concomitant flattening of the redox curve at a relatively oxidized redox potential.

### Redox/auxin signaling and root development

While our work points to a central role for redox gradients in the structure and functioning of the root meristem, exactly how redox signaling affects root meristem development and maintenance is not known. Kerk and Feldman (1995) suggested that the redox status of the maize QC was central to root meristem structure and activity, and proposed a model linking auxin and redox status at the root meristem. Here, we show that a salt-associated change in redox status at the root tip is paralleled by a change in the distribution of auxin transport proteins, thereby supporting a possible link between redox signaling and auxin, and in agreement with earlier work of Pasternak et al. (2002) and Bashandy et al. (2010). Using Arabidopsis mutated in key redox-regulating enzymes these workers showed a decrease in polar auxin transport and thereby concluded that redox status (specifically, glutathione homeostasis) plays a role in root development by affecting auxin signaling, a conclusion supported and extended by Yu et al. (2013) who proposed that glutathione redox status affects the root apical meristem, in part, through an auxin/PLETHORA (PLT)- dependent pathway. More recently, Iglesias et al. (2014) showed that salinity leads to a suppression of auxin signaling by triggering “the stabilization of Aux/IAA repressors,” leading these workers to propose that salt stress signals are integrated into plant development by an “ROS-auxin crosstalk.” In this context, the salt-induced alterations to auxin transporters, which we have observed, might be part of a mechanism to suppress auxin signaling and thereby enhance tolerance to salinity. In this regard too the observations of Sun et al. (2008) that salt treatment (150 mM) results in a decrease in PIN2 abundance, and the recent work of Liu et al. (2015) that salt stress downregulates the expression of *PIN* genes, provides added evidence that salt may affect auxin distribution in the root by affecting the levels/operation of auxin transporters. This downregulation in *PIN* gene expression may be related to the modest increase in the expression of an auxin-responsive promoter (*DR5GUS*) in the QC, which both we and Chávez-Avilés et al. (2013) have observed. Interestingly, Liu et al. (2015) also report that salt treatment affects the level of expression of DR5 in the root tip, but they link salt treatment to a modest decrease, rather than an increase, as we report, in DR5-GFP expression. So while we do not yet know many of the specifics of the manner in which redox influences auxin status in the root (and likely, vice versa as well), evidence suggests that salt-induced changes in redox status in roots can influence root meristem maintenance, at least in part, by affecting auxin transport. Worth mentioning with regard to the work of Kerk and Feldman (1995) and Jiang et al. (2003) are their observations that the QC was the *most* oxidized population of cells (in maize roots), a result which differs from what we here report for Arabidopsis, in which the QC maintains a relatively reduced status. The resolution of this apparent contradiction may rest on the fact that Jiang et al. (2003) used a redox-reporter sensitive to a rather narrow range of redox species, whereas for the current effort the roGFP has a broader range and therefore likely provides a more complete picture of overall redox status (Jiang et al., 2006).

In summary, we report the redox status of specific populations of cells which comprise the Arabidopsis root tip, and as well explore the effects of salt (NaCl) on redox profiles. From the complex redox responses of roots to salt, a number of general observations can be made. First, roots respond rapidly to exposure to salts by shifting their overall redox status to more oxidized values, which initially come to be more or less the same along the entire length of the root tip, thereby eliminating any differences in redox potentials between the different regions (i.e., RC, QC, PM, TZ, EZ). Secondly, depending on the salt concentration (<100 mM), Arabidopsis roots are able to restore redox levels and patterns to control values. However, as salt levels increase (between 100 and 150 mM), Arabidopsis roots are less able to moderate their redox status to more reduced, control values. Finally, we provide evidence that salt stress affects root development and maintenance, in part, through changes in redox/auxin transport.

## Author contributions

All authors contributed equally to the research. The paper was written by LF and KJ.

### Conflict of interest statement

The authors declare that the research was conducted in the absence of any commercial or financial relationships that could be construed as a potential conflict of interest.

## Supplementary material

Supplementary Figure 1 **(A,B)** Redox profiles for control roots grown continuously on agar **(A)** for 3, 6, or 9 days or on agar plus immersion **(B)** for 3, 6, 12, or 24 h. Each curve in **(A)** represents the average of 16 roots, with accompanying standard deviations, and for **(B)** each curve is the average of eight roots, with accompanying standard deviations. For each treatment the control curve is an average of 16 roots at the same time point.

Supplementary Figure 2 **(A–D)** Redox profiles for immersed, salt-treated (150 mM NaCl) roots. **(A)** 3 h salt treatment; **(B)** 6 h salt treatment; **(C)** 12 h salt treatment; **(D)** 24 h salt treatment. Each curve represents the average of eight roots, with accompanying standard deviations. For each treatment the control curve is an average of eight roots at the same time point.

Supplementary Figure 3 **(A–H)** Individual Redox profiles of Arabidopsis primary roots grown on solid medium and treated for extended periods (3–9 days) with various concentrations of NaCl (50–150 mM). This figure shows the individual curves, with standard deviations, used to generate the summary redox profiles in Figures 3A–C.
