# Supplementary figures and images for: Salt Stress Affects the Redox Status of Arabidopsis Root Meristems
Source: Front Plant Sci. 2016 Feb 8;7:81. doi: 10.3389/fpls.2016.00081 (PMC4744855; doi:10.3389/fpls.2016.00081)

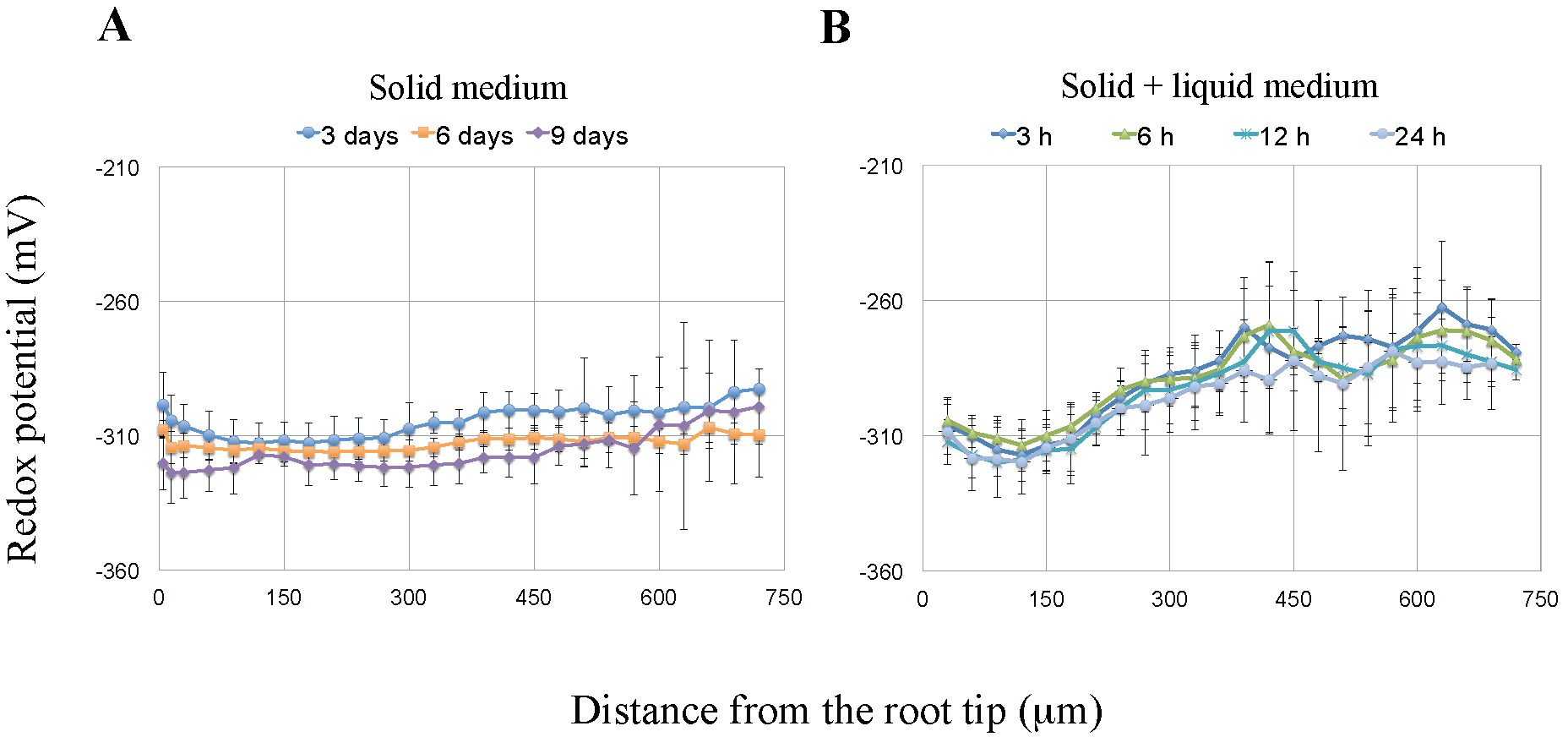

Supplement: Supplementary Figure 1 — (A,B) Redox profiles for control roots grown continuously on agar (A) for 3, 6, or 9 days or on agar plus immersion (B) for 3, 6, 12, or 24 h. Each curve in (A) represents the average of 16 roots, with accompanying standard deviations, and for (B) each curve is the average of eight roots, with accompanying standard deviations. For each treatment the control curve is an average of 16 roots at the same time point. [file Image1.TIF]

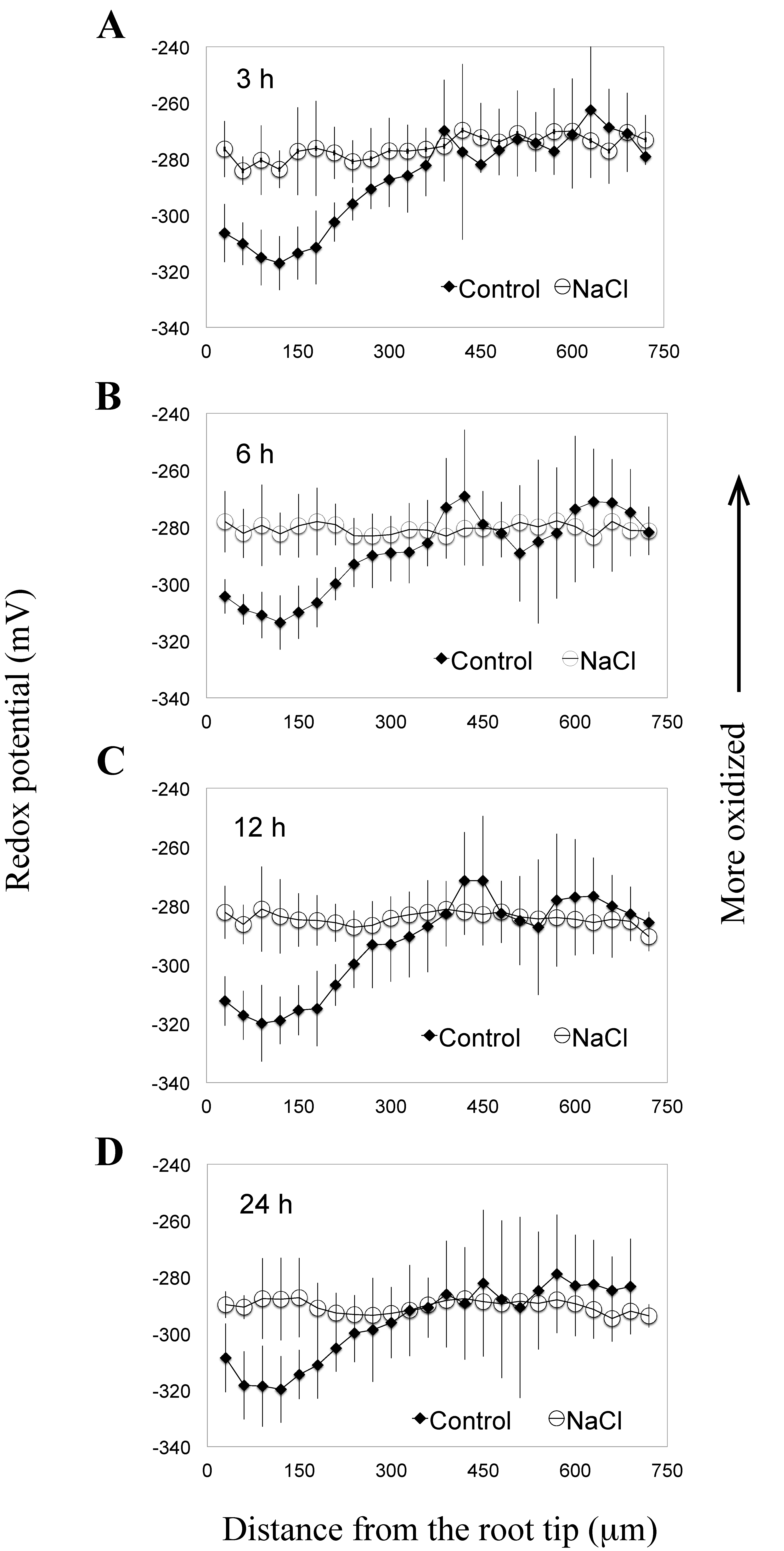

Supplement: Supplementary Figure 2 — (A–D) Redox profiles for immersed, salt-treated (150 mM NaCl) roots. (A) 3 h salt treatment; (B) 6 h salt treatment; (C) 12 h salt treatment; (D) 24 h salt treatment. Each curve represents the average of eight roots, with accompanying standard deviations. For each treatment the control curve is an average of eight roots at the same time point. [file Image2.TIF]

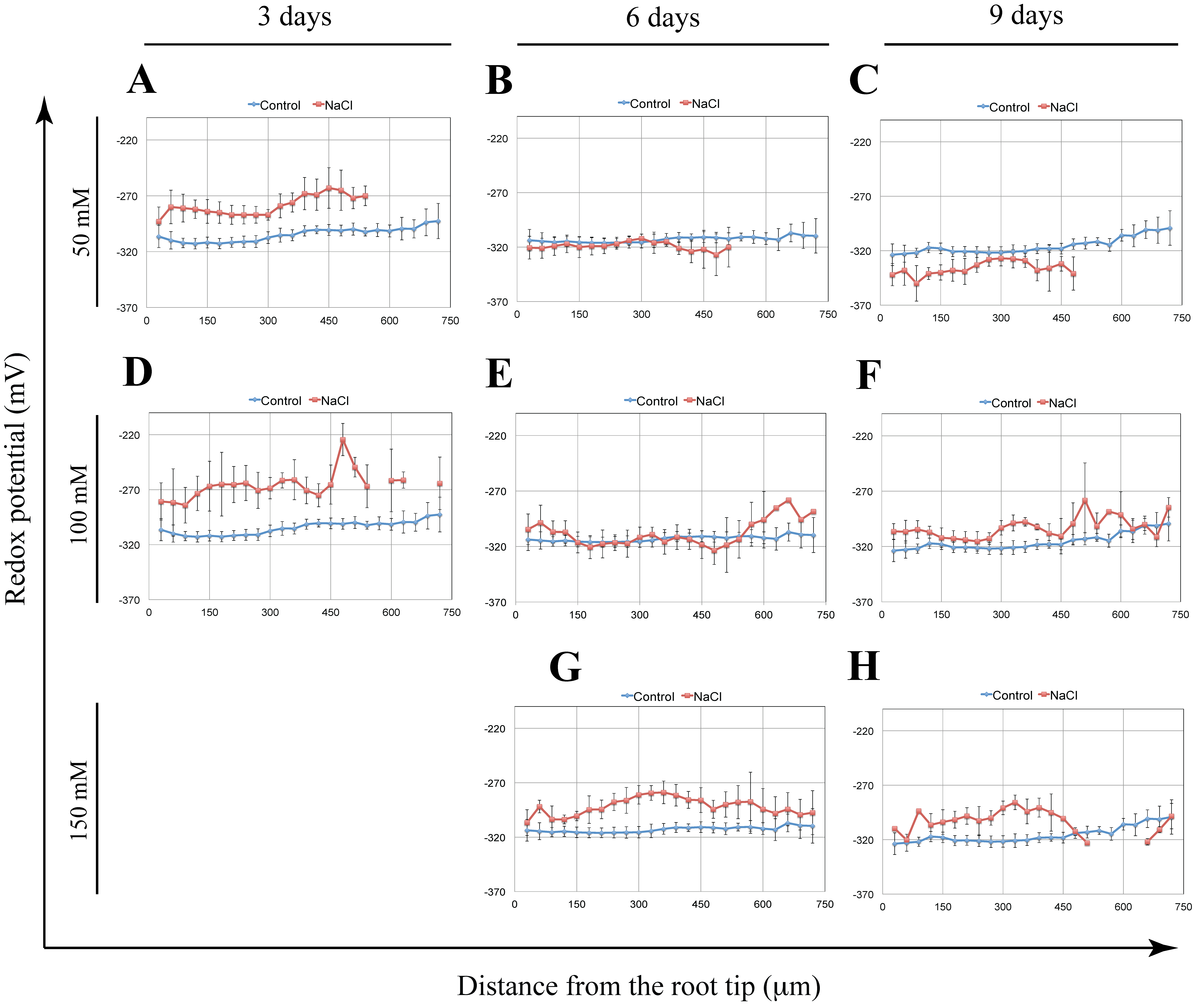

Supplement: Supplementary Figure 3 — (A–H) Individual Redox profiles of Arabidopsis primary roots grown on solid medium and treated for extended periods (3–9 days) with various concentrations of NaCl (50–150 mM). This figure shows the individual curves, with standard deviations, used to generate the summary redox profiles in Figures 3A–C. [file Image3.TIF]
